# Identification of potential natural inhibitors of the receptor-binding domain of the SARS-CoV-2 spike protein using a computational docking approach

**DOI:** 10.5339/qmj.2021.12

**Published:** 2021-03-12

**Authors:** Shilu Mathew Mathew, Fatiha Benslimane, Asmaa A. Althani, Hadi M. Yassine

**Affiliations:** ^1^Biomedical Research Center, Qatar University, Doha, Qatar; ^2^Department of Biomedical Sciences, College of Health Sciences-QU Health, Qatar University, Doha, Qatar E-mail: hyassine@qu.edu.qa

**Keywords:** Virtual screening, Molecular docking, Antiviral drugs, Computational docking, SARS-CoV-2, Spike protein

## Abstract

**Background:** The novel severe acute respiratory syndrome coronavirus 2 (SARS-CoV-2) is the only zoonotic-origin CoV to reach the pandemic stage, to which neither an effective vaccine nor a specific therapy is available. The spike glycoprotein harbors the receptor-binding domain (RBD) that mediates the virus's entry to host cells. This study aimed to identify novel inhibitors that target the spike protein's RBD domain through computational screening of chemical and natural compounds. **Method:** The spike protein was modeled from the recently reported electron microscopy protein structure (PDB ID: 6VSB) and the previously described SARS-CoV protein structure (PDB ID: 6ACD and 6ACJ). Virtual lab bench CLC Drug Discovery was used to computationally screen for potential inhibitory effects of currently prescribed drugs (n = 22), natural antiviral drugs (n = 100), and natural compounds (n = 35032). Quantitative Structure-Activity Relationship (QSAR) studies were also performed to determine the leading binders known for their antiviral activity. **Results:** Among the drugs currently used to treat SARS-CoV2, hydroxychloroquine and favipiravir were identified as the best binders with an average of four H-bonds, with a binding affinity of − 36.66 kcal/mol and a minimum interaction energy of − 6.63 kcal/mol. In an evaluation of antiviral compounds, fosamprenavir and abacavir showed effective binding of five H-bonds, with an average binding affinity of − 18.75 kcal.mol^− 1^ and minimum interaction energy of − 3.57 kcal/mol. Furthermore, screening of 100 natural antiviral compounds predicted potential binding modes of glycyrrhizin, nepritin, punicalagin, epigallocatechin gallate, and theaflavin (average binding affinity of − 49.88 kcal/mol and minimum interaction energy of − 4.35 kcal/mol). Additionally, the study reports a list of 25 natural compounds that showed effective binding with an improved average binding affinity of − 51.46 kcal/mol. **Conclusions:** Using computational screening, we identified potential SARS-CoV-2 S glycoprotein inhibitors that bind to the RBD region. Using structure-based design and combination-based drug therapy, the identified molecules could be used to generate anti-SARS-CoV-2 drug candidates.

## Introduction

SARS-CoV-2 is the causative agent of coronavirus disease 2019 (COVID-19), which emerged in Wuhan, China in December 2019.^1,2^ Infection with SARS-CoV-2 could be asymptomatic or result in mild flu-like symptoms in approximately 80% of patients. However, elderly COVID-19 patients and those with underlying health conditions may suffer from severe illnesses, such as pneumonia and acute respiratory failure. In this category of patients, COVID-19 could be fatal.^2–4^ As of mid-June 2020, SARS-CoV-2 had infected over 7.5 million people in 188 countries and territories and caused >450 thousand fatalities.^5^ The virus has a very high infectivity rate, making it more contagious than other coronaviruses (CoV), including the severe acute respiratory syndrome coronavirus 1 (SARS-CoV-1) and Middle East respiratory syndrome coronavirus (MERS-CoV).^6,7^ Due to its widespread distribution, COVID-19 was declared a pandemic by the World Health Organization (WHO) on March 11, 2020.^8^ Despite continuous efforts to contain the pandemic, resistant strains might sporadically emerge and spread worldwide considering the wide use of inhibitor drugs for COVID-19. Current treatments rely on already existing antiviral repurposed drugs in combination with antibiotics for secondary infection treatment.^9^ Hence, the development of effective prophylactics and vaccines against SARS-CoV-2 remains urgent.^10–12^

Antiviral therapies act either directly on the virus to inhibit its replication or on the immune system to enhance host defense mechanisms. Specifically, therapeutics that act directly on the virus target either structural proteins required for infection or non-structural proteins (e.g., polymerase) required for replication.^13^ The viral attachment protein (surface glycoprotein) is considered the key that unlocks host cells. This critical mechanism allows the virus to successfully invade its host. Hence, surface glycoproteins serve as targets for the development of therapeutics and vaccines. In the case of SARS-CoV-2, S glycoprotein plays the most critical role in viral attachment, fusion, and entry. The SARS-CoV-2 spike protein receptor-binding domain (S-RBD) recognizes the human receptor angiotensin-converting enzyme 2 (ACE2), triggering fusion between the virus and host membranes.^14,15^ Therefore, chemicals that target the RBD could potentially block viral entry and thus be used as an effective COVID-19 therapy.^14^

In this study, we used virtual screening methods to model the SARS-CoV-2 S glycoprotein and screen a library of current drugs available on the market, natural antiviral compounds and natural compounds, for potential binding activities to S-RBD.

## Methods

### Homology modeling of SARS-CoV-2 spike protein

Homology modeling of the SARS-CoV-2 spike sequence was conducted using Modeller 9.24. Full-length models built by Modeller were based on multiple alignments of the top three selected templates: protein database (PDB) ID 6ACJ, resolution 4.2 Å: SARS-CoV spike glycoprotein and ACE2 complex;^16^ PDB ID 6ACD, resolution 3.9 Å, SARS-CoV spike glycoprotein and ACE2 complex-ACE2-free conformation with one RBD in the up conformation;^16^ and PDB ID 6VSB, resolution 3.46 Å; prefusion SARS-CoV-2 spike glycoprotein with a single receptor-binding domain up.^17^ The modeled structure with the lowest free energy was energy minimized by using ModRefiner Server,^18^ which follows a two-step procedure for constructing a full-atom model. The first step builds the backbone for the available C-alpha and performs energy minimization to improve the quality. The second step adds side-chain atoms from a rotamer library and conducts energy minimization of both side chains and backbone conformations.^18^

### Modeled SARS-CoV-2 spike structure validation

The final refined SARS-CoV-2 spike model was validated using PROCHECK (Structural Analysis and Verification Server) to calculate Ramachandran's plot.^19^ Superpose (Version 1.0) was used to analyze the energy criteria of SARS-CoV-2 spike modeled proteins with the 3D template structure ^20^ and to calculate the root-mean-square deviation (RMSD) value with the template.^20^

### Protein preparation

PDB structures of the SARS-CoV-2 spike model were imported into the CLC Drug Discovery Workbench. These imported proteins were assigned with polar hydrogen for appropriate treatment of electrostatic docking.^21^ The assigned structures were named Protein Data Bank, Partial Charge (Q), and Atom Type (T) (PDBQT) files to be used as coordinate files including atomic partial charges and atom types. The PDBQT file was further refined with the H-bond assessment (neutral pH, water orientations), and Merck molecular force field (MMFF) 94 was used as an energy minimization force field.^21^ A grid for the processed protein was generated using the site around the selected residues targeting the spike protein.

### Ligand preparation

The structure of the 22 available drug molecules being used for SARS-CoV-2 treatment and other available drugs used for treating other viruses were obtained from PubChem.^22^ (Supplementary Table 1). The 2D chemical structure of 100 natural molecules with antiviral properties was also drawn using ACD Chemsketch.^23^ (Supplementary Table 2). Additionally, 35,032 natural compounds were retrieved from natural product activity and species source database (NPASS) and screened as potential S-RBD binders.^24^ Ligand preparation was done using MMFF94 geometry operation, and the Gasteiger charge was calculated at pH 7.0.^25^ Ligand preparation was applied using Balloon, a freely available 3D structures program generator.^26^ All the selected structures were imported in Spatial Data File (SDF) strings format in CLC Drug Discovery Workbench.

### Pharmacokinetics structure-activity relationship

The physicochemical properties and relation of the best binders with biological activity were evaluated.^27^ The Quantitative Structure-Activity Relationship (QSAR) predicts the compound's expected biological response according to its chemical structure.^27^ Virtual models for property evaluation of chemicals within a Global Architecture (VEGA-QSAR) is an independent java-based web program that predicts QSAR properties and screens similar compounds in a read-across strategy. Mutagenicity (Ames test) CONSENSUS model 1.0.3, Carcinogenicity model (CAESAR) 2.1.9, Developmental Toxicity model (CAESAR) 2.1.7, Skin Sensitization model (CAESAR) 2.1.6, Hepatotoxicity model (IRFMN) 1.0.0, Ready Biodegradability model (IRFMN) 1.0.9, and LogP Prediction [Log Units] models were evaluated by publicly well-known open and commercial QSAR prediction software package VEGA.^28^ SDF files were used as input formats of the ligands’ 2D structures from PubChem.^27^

### Sars-cov-2 S-rbd

The selected drugs were screened for their binding affinity to the S-RBD site. According to the S-ACE2 complex structure (6VSB),^16,17^ the Surface domain 1 (SD1) of RBD spans amino acids AA 326 to 559 and contains a core and a receptor-binding motif that mediates contact with ACE-2.^15^ We also investigated interactions at potential N-linked glycosylation (165, 234, 282, 709, 717, 801, 1098, and 1134) sites, O-linked glycosylation sites (residues 318, 319, 323, 325, 330, 357, 673, 678, and 686), 10 hot spot residues (390, 426, 429, 431, 455, 473, 483, 492, 494, and 495) that are known to reduce binding to ACE2 significantly, and specific residues (393, 442, 472, 479, 480, 487 and 493) that appear to provide favorable interactions with human ACE2.^29^

### Docking protocol

Molecular docking studies for ligands were performed using CLC Drug Discovery Workbench. ^26^ A PDBQT file was prepared for ligands via AMBER force field by adding hydrogen atoms. The docking wizard was used to apply the default MolDock optimizer algorithm with the following docking parameters: maximum iterations = 2000; number of runs = 200; RMSD thresholds for similar cluster poses = 1.00, crossover rate = 0.90, and scaling factor = 0.50.^30^ The best-ranked compounds were selected on the basis of hydrogen interactions and docking scores and were visualized using the CLC Drug Discovery Visualization tool.^31,32^.

## Results

### General characteristics of modeled SARS-CoV-2 spike protein in comparison with SARS-CoV-1

**Figure 1.**
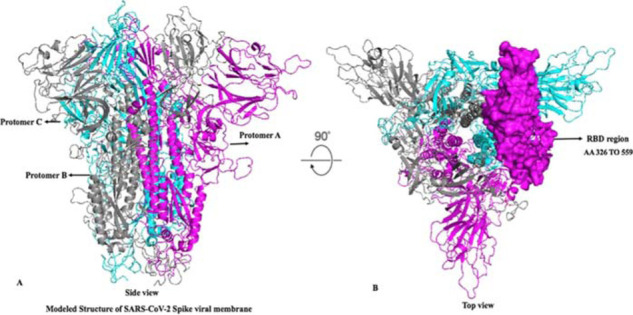
(A) Schematic of modeled SARS-CoV-2 S trimeric structure colored by protomer (Chain A in pink, Chain B in gray, and Chain C in cyan). Side views of the prefusion structure of the SARS-CoV-2 Spike protein with a single RBD in the up conformation. (B) Top view of the prefusion structure of the SARS-CoV-2 Spike protein. RBD protomer is shown as surface colored corresponding to the schematic color in A

We first constructed first-generation homology models of the SARS-CoV-2 spike protein using the surface glycoprotein sequence QHD43416.1. Our initial BLAST analysis indicated that the virus spike protein was most closely related to a clade SL-CoVZXC21 of bat SARS-like coronaviruses (sequence ID: AVP78042.1). Since no high-resolution spike X-ray structure was available, we constructed a homology model of the spike protein with the template protein, which showed 76.47% similarity to the protein structure of PDB ID 6ACD and 6ACJ available in the PDB Bank (www.rcsb.org). As of recently, the cryo-electromagnetic (EM) structure of the SARS-CoV-2 S trimer in the prefusion conformation with an N-acetyl-D-glucosamine (PDB ID: 6VSB) was also available, allowing us to consider this structure in the remodeling of the protein structure. We performed multiple sequence alignment of the three proteins PDB ID 6ACD, 6ACJ, and 6VSB (1-1212 AA) as an input and thereby considered them a template homology model. Surface glycoprotein QHD43416.1 shared 95% query coverage and 76.47% identity to 6ACD and 6ACJ but shared 98% query coverage and >95% identity to 6VSB. A python-based protein model was constructed using Modeller v9.12, which constructs a 3D model by satisfying spatial constraints with respect to multiple sequences, structure alignment, clustering, and flexibility. The Ramachandran plot using structural analysis and verification server (SAVS) PROCHECK version 4 evaluated the best model from the above. The Ramachandran plot for the modeled SARS-CoV-2 spike protein exhibited only 98.8% AA residues in highly favorable regions, 0.8% and 0.4% in allowed and disallowed regions, respectively. Figure 1A denotes the modeled SARS-CoV-2 S trimeric structure colored by protomer, and Figure 1B illustrates the top view of the modeled SARS-CoV-2 S trimeric structure with surface representation for the S-RBD fragment positioned at AA 326 to 559.

### Selection of the best binders among the currently prescribed drugs

**Figure 2.**
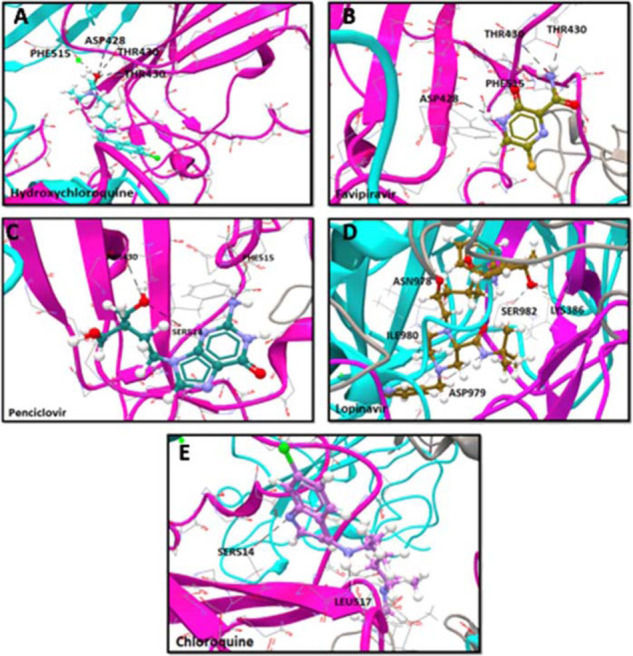
Computational docking confirmation of the five best binders of currently prescribed drugs to the RBD region of the SARS-CoV-2 spike protein. Analysis was performed with CLC Drug Discovery Workbench while considering the important parameters including BA, IE, and HB interactions.The top panel depicts the binding characteristics of five molecules to their targets. All compounds were retrieved from PubChem, including (A) Hydroxychloroquine, (B) Favipiravir, (C) Penciclovir, (D) Lopinavir, and (E) Chloroquine, and are shown as 3D structures. Modeled SARS-CoV-2 spike protein protomers A, B, and C are represented as pink, gray, and cyan ribbon-like structures. The anchored HB between the compound and RBD epitopes are shown in black.

To refine the hit compounds, all initially prescribed compounds for SARS-CoV-2 treatment (n = 12) were docked into the binding site S-RBD region using the CLC Drug Discovery Workbench.^33^ The docked compounds were top-ranked for binding to the RBD region according to the following criteria: low interaction energy (IE), low binding affinity (BA), and high hydrogen bonding. The IE and BA were calculated for each hit compound after energy minimization and reported in kcal/mol unit. Of the 12 currently prescribed drugs, five exhibited the maximum average H-bond interactions ^3^, BA score ( − 6.63 kcal/mol), and low IE ( − 36.66 kcal/mol) to the RBD region of the spike protein. These lead molecules include hydroxychloroquine, favipiravir, penciclovir, lopinavir, and chloroquine. The binding characteristics of the 12 currently available drug molecules and their interactions are summarized in Table 1. The docking conformations of the abovementioned lead binders are illustrated in Figure 2. Hydroxychloroquine and favipiravir exhibited a maximum of four H-bonds with a minimum BA score ( − 45.44 kcal/mol) (Figure 2 & 2B). Both hydroxychloroquine and favipiravir interacted with the same RBD residues ASP428, THR430 ^2^, and PHE515, with a minimum average IE score of − 7.99 kcal/mol and a BA of − 45.44 kcal/mol (Figure 2A & 2B). On the other hand, penciclovir exhibited second-best atomic interaction with SD1 residues THR430, SER574, PHE515 with minimum IE of − 5.39 kcal/mol and a BA of − 35.86 kcal/mol (Figure 2C). It was also noted that lopinavir extended only two H-bonds with RBD region LYS386 ^2^, but in the midst of its interaction, it extended H-bonds in the SD2 regions of the spike protein anchoring with residues ASN978, ILE980, ASP979, and SER982 (IE of − 6.45 kcal/mol and a BA of − 25.82 kcal/mol.) (Figure 2D). The fifth potential binder chloroquine exhibited only two H-bonds with the RBD region (SER514 and LEU517) with a minimum IE of − 3.56 kcal/mol and a BA of − 25.82 kcal/mol (Figure 2E). Similarly, nafamostat and nitazoxanide extended very few interactions but with maximum positive IE and BA scores. From the docking conformations, it was also observed that Chlorphenamine did not show any interaction within the binding pockets of the RBD region. We observed that none of the listed compounds interacted at N-linked vs. O-linked glycosylation sites and the RBD region's hot spot residues.

### Screening of best binders among the antiviral drugs

**Figure 3.**
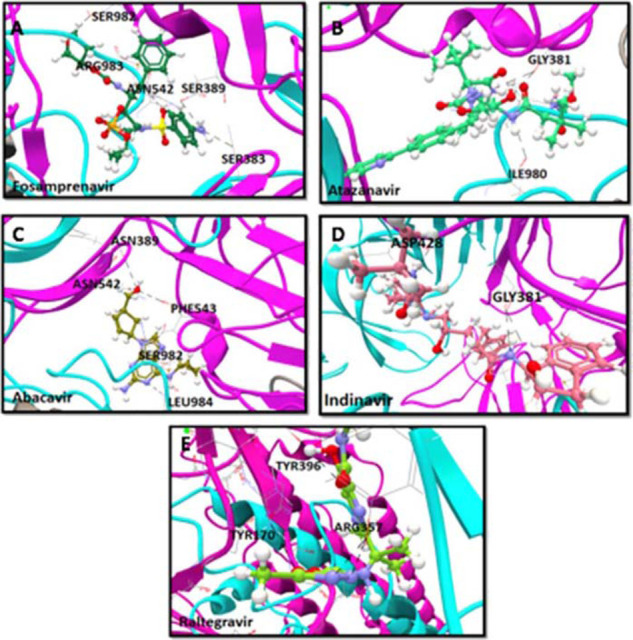
Computational docking confirmation of the five best antiretroviral binding compounds to the RBD region of the SARS-CoV-2 spike protein. Analysis was performed with CLC Drug Discovery Workbench while considering the important parameters, including BA, IE, and HB interactions.The top panel depicts the binding characteristics of five molecules to their target. All compounds were retrieved from PubChem, including (A) Fosamprenavir, (B) Atazanavir, (C) Abacavir, (D) Indinavir, and (E) Raltegravir and are shown as 3D structures. Modeled SARS-CoV-2 spike protein protomer A, B, and C are represented as pink, gray and cyan ribbon-like structures. The anchored HB between the compound and RBD epitopes are shown in black.

A molecular docking approach was employed to screen 10 antiviral drugs for binding activity to SARS-CoV-2 modeled S-RBD. The lead biding molecules with maximum interactions and minimum IE and BA scores included fosamprenavir, atazanavir, abacavir, indinavir, and raltegravir. The binding characteristics of the 10 molecules are summarized in Table 2. The docking conformations of the abovementioned lead binders are illustrated in Figure 3. Acutely few energetically favorable interactions were observed with antiviral compounds in close contact with the RBD region. Fosamprenavir and abacavir exhibited a maximum number of three H-bond interactions with the RBD region and extensive interaction with other residues in the spike (Figure 3A & 3B). In detail, fosamprenavir interacted with SER383, ASP389, ASN542, SER982, and ARG983 in the SD1 and SD2 regions with minimum IE of − 5.66 kcal/mol and a BA of − 22.66 kcal/mol (Figure 3A). In comparison, abacavir interacted with PHE543, ASP389, ASN542, LEU984, SER982 with minimum IE of − 6.88 kcal/mol and a BA of − 18.85 kcal/mol (Figure 3B). Atazanavir and indinavir exhibited interactions with residue GLY381 ^2^ with an average IE of − 5.86 kcal/mol and a BA of − 18.79 kcal/mol (Figure 3C & 3D). Furthermore, raltegravir anchored with ARG357 ^2^, TYR396, and TYR170 with a BA of − 15.85 kcal/mol. Apart from their interaction with each other, antiviral compounds interacted and formed complexes with other residues outside of the RBD binding site in the spike protein (Figure 3E). Moreover, among the selected antiviral drugs in this study, only raltegravir anchored at the O-linked glycosylation site ARG357, while there was no interaction with hot spot residues in the RBD region.

### Screening of the best binders among natural antiviral compounds

**Figure 4.**
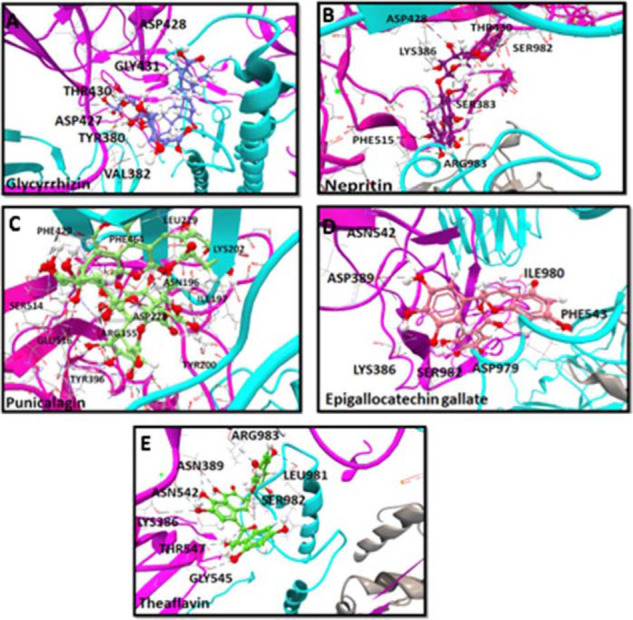
Computational docking confirmation of the five best binders of antiviral natural compounds to the RBD region of the SARS-CoV-2 spike protein. Analysis was performed with CLC Drug Discovery Workbench while considering the important parameters, including BA, IE, and HB interactions.The top panel depicts the binding characteristics of five molecules to their target. All compound were retrieved from PubChem, including (A) Glycyrrhizin, (B) Nepritin, (C) Punicalagin, (D) Epigallocatechin, gallate, and (E) Theaflavin, and are shown as 3D structures. Modeled SARS-CoV-2 spike protein protomers A, B, and C are represented as pink, gray, and cyan ribbon-like structures. The anchored HB between the compound and RBD epitopes are shown in black.

We next screened 100 natural antiviral compounds (Supplementary Table 2) and selected the 10 best binders that exhibited a maximum number of H-bonds and minimum IE and BA scores. These 10 lead binders included glycyrrhizin, nepitrin, punicalagin, epigallocatechin gallate (EGCG), theaflavin, silibinin, galuteolin, 7-galloytriacetone, procyanidin, and catechin. The docking conformations of the 10 lead antiviral natural binders are illustrated in Figure 4. The binding characteristics of the 12 molecules are summarized in Table 3. Among the top 10 binders, glycyrrhizin topped with 12 H-bond interactions, specifically blocking the RBD binding pockets (Figure 4A). Besides glycyrrhizin, punicalagin also formed significant interactions by exhibiting 23 H-bond interactions in the RBD SD1 region as well as extending to another SD2 part of the spike protein (Figure 4C). Notably, glycyrrhizin displayed atomic interactions with residues VAL382, TYR380 ^2^, THR430 ^4^, GLY431, ASP428 ^2^, and ASP427 ^2^ with a minimum IE of − 6.32 kcal/mol and a BA of − 42.55 kcal/mol (Figure 4A). Analysis of the punicalagin interaction showed H-bond interactions with residues PHE429, DER514 ^2^, GLU516 ^2^, TYR396, ARG355, PHE464, TYR200, ILE197, LYS202, ASN196, LEU229, and ASP228 ^2^ with minimum IE of − 5.55 kcal/mol and a BA of − 50.34 kcal/mol. Apart from glycyrrhizin, the rest of the best binders extended interactions with other regions of the spike protein. Nepitrin ranked as the second-best binder, extending 10 H-bond interaction with residues ASP428, THR430 ^2^, PHE515, LYS386, ^2^, ARG983, SER383, and SER982 ^2^ with minimum IE of − 6.32 kcal/mol and a BA of − 47.85 kcal/mol (Figure 4B). EGCG, one of the potential antiviral compounds, also exhibited potential inhibition activity as it extended 12 H-bonds with AA residues LYS386 ^2^, ASP389 ^3^, ASN542 ^2^, PHE543, SER982, ILE980, and ASP979 ^2^ with minimum IE of − 6.32 kcal/mol and a BA of − 52.15 kcal/mol (Figure 4D). Theaflavin showed the least BA, compared with other antiviral compounds with the energy of − 56.55 kcal/mol and anchored with residues in GLY545, THR547, LYS386, ASN389^4^, ASN542, ASN978, SER982^2^, LEU981, and ARG983 (Figure 4E). None of the natural antiviral compounds interacted with the potential glycosylation N-linked and O-linked sites, except glycyrrhizin interaction with GLY431 and punicalagin interaction with hot spot residue PHE429.

### Screening of the best binders among natural compounds

**Figure 5.**
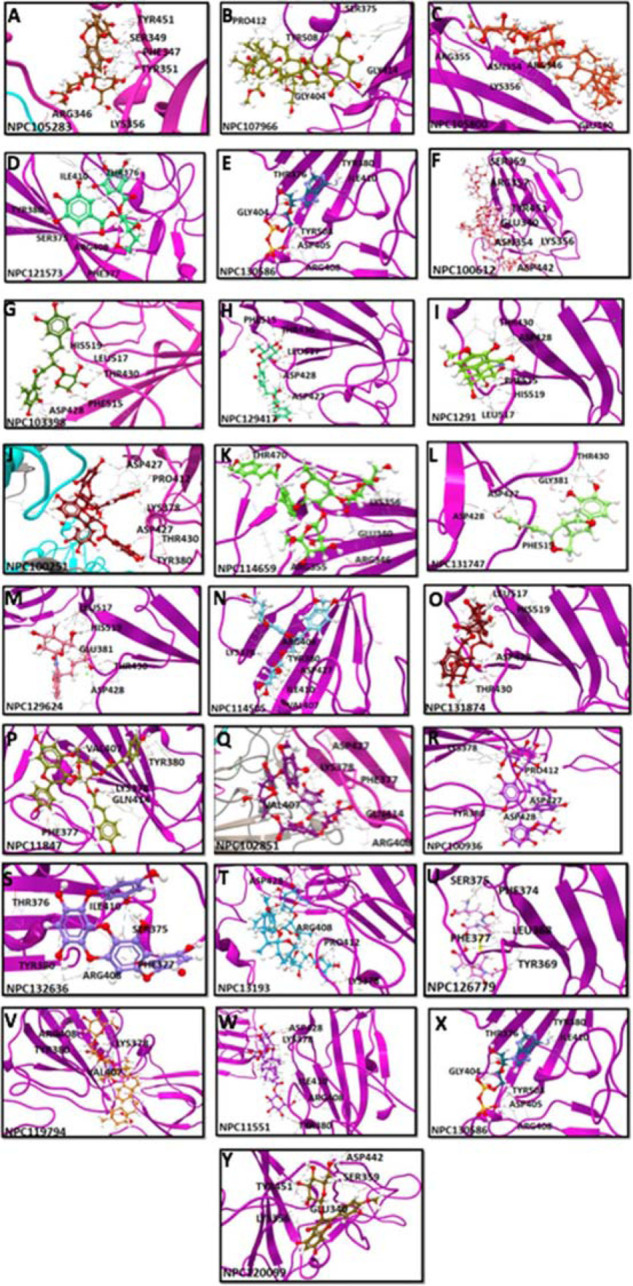
Computational docking confirmation of the 25 best binders of natural compounds to the RBD region of the SARS-CoV-2 spike protein. Analysis was performed with CLC Drug Discovery Workbench while considering the important parameters, including BA, IE, and HB interactions.The top panel depicts the binding characteristics of 25 molecules to their targets. All compounds were retrieved from PubChem, including (A)NPC105283, (B) NPC107966, (C) NPC105800, (D) NPC121573, (E) NPC130586, (F) NPC100612, (G) NPC103398, (H) NPC129417, (I) NPC1291, (J) NPC100251, (K)NPC114659, (L) NPC131747, (M) NPC129624, (N) NPC114505, (O) NPC131874, (P) NPC11847, (Q) NPC102851, (R) NPC100936, (S) NPC132636, (T) NPC13193, (U) NPC126779, (V) NPC119794, (W)NPC11551, (X)NPC130536, and (Y) NPC120099, and are shown as 3D structures. Modeled SARS-CoV-2 spike protein protomers A, B, and C are represented as pink, gray, and cyan ribbon-like structures. The anchored HB between the compound and RBD epitopes are shown in black.

A virtual screening strategy was used to screen candidate inhibitors of the RBD region in a compound library containing 35,032 natural compounds by using in CLC Drug Discovery Workbench. Following virtual screening, 25 natural compounds with maximum numbers of H-bonds and minimum IE and BA scores were selected as candidate inhibitors. These 25 lead binders included NPC105283, NPC107966, NPC105800, NPC121573, NPC130586, NPC100612, NPC103398, NPC129417, NPC1291, NPC100251, NPC114659, NPC131747, NPC129624, NPC114505, NPC131874, NPC11847, NPC102851, NPC100936, NPC132636, NPC13193, NPC126779, NPC119794, NPC11551, NPC130536, and NPC120099. The docking conformations of the 25 natural binders are illustrated in Figure 5. The binding characteristics of the 25 molecules are summarized in Table 4. Notably, the 20 best binders were ranked as per the number of H-bonds formed, and all the best binders efficiently anchored specifically within the RBD regions. Two of the top compounds, NPC105283 and NPC107966, extended 11 H-bond interactions with average IE of − 2.77 kcal/mol and a BA of − 56.6 kcal/mol (Figure 5A & 5B). NPC105283 anchored with TYR351, SER349^3^, PHE347, ARG346^3^, LYS356, and TYR451 (Figure 5A). NPC107966 interacted with PRO412, TYR508^2^, GLY404, GLN414, LYS378^2^, and SER378^3^ (Figure 5B). NPC105800, NPC121573, NPC130586, and NPC100612 formed 10 H-bonds with average IE of − 2.31 kcal/mol and a BA of − 51.29 kcal/mol (Figure 5C–5F). NPC103398, NPC129417, and NPC1291 formed nine H-bonds with an average IE of − 3.11 kcal/mol and a BA of − 51.34 kcal/mol (Figure 5G–5J). NPC100251, NPC114659, NPC131747, NPC129624, and NPC114505 formed eight H-bonds with average IE of − 3.25 kcal/mol and a BA of − 50.90 kcal/mol (Figure 5K–5O). NPC102851, NPC100936, NPC132636, NPC13193, and NPC126779 formed seven H-bonds with an average IE of − 5.05 kcal/mol and a BA of − 52.46 kcal/mol (Figure 5P–5U). NPC119794, NPC11551, NPC1030586, and NPC120099 formed six H-bonds with an average IE of − 4.11 kcal/mol and a BA of − 51.38 kcal/mol (Figure 5T–5Y). Moreover, these screened natural inhibitors did not interact with any of the potential glycosylation or hot spot residues but significantly occupied the RBD SD1 region of the new anti-SARS-CoV-2 spike protein, compared with currently prescribed drugs and natural antiviral compounds.

### VEGA-QSAR profiling of screened products

All the best binders were then screened through the QSAR model for their biological activities, including mutagenicity, sensitivity, developmental toxicity, biodegradability, hepatotoxicity, and logP prediction. The results evaluated by QSAR models denoted that 90% of the lead binders were non-mutagens, non-toxicants, non-carcinogens, and non-sensitizers (Supplementary Table 3). Out of the five top antiviral compounds, theaflavin was a positive predictor in the carcinogenicity, developmental toxicity, and hepatotoxicity models. Likewise, punicalagin, which formed significant interactions with RBD, had a positive prediction for the mutagenicity, toxicity, and hepatotoxicity models. Moreover, the only antiviral compound was predicted to be readily biodegradable, compared with the other compounds. Among the natural compounds, 90% of the lead binders were non-mutagens, non-carcinogens, and non-sensitizers. However, approximately 85% of the natural compounds were predicted to be toxic, followed by non-readily biodegradable. Predicted log *P*-values of the lead binders ranged from 0.04 to 9.15 log units. Predicted properties of the best binders to the RBD region for various models are summarized in (Supplementary Table 4).

## Discussion

Both SARS-CoV utilize the ACE2 to invade host cells.^17,34^ Hence, intervention strategies that block SARS-CoV S-RBD binding to its receptor would prevent infection. Here we employed a structure-based approach to screen and select drugs and natural compounds with RBD binding activity, identifying them as potential inhibitors for SARS-CoV-2 infection.

To this end, we initially constructed homology models of the SARS-CoV-2 spike protein. Since no high-quality spike X-ray crystallography structure was available, we selected a template SARS-CoV-2 spike protein with PDB ID: 6ACD and 6ACJ, which showed 76.47% similarity to the QHD43416.1 sequence. The Cryo-EM structure of the SARS-CoV-2 S trimer was resolved recently, allowing us to include its PDB structure in the remodeling of the spike protein. The best dockable model from the above was verified by the Ramachandran plot and was considered for molecular docking by using the CLC Drug Discovery Workbench. We employed docking analysis of currently prescribed and antiviral compounds for COVID-19 (n = 22), 100 natural antiviral molecules, and 35032 natural compounds from the NPASS database.^24^ Compounds that formed the highest number of H-bonds, with a minimum number of IE and BA, were selected as the best potential binders. We also investigated the interaction at the functional glycosylation and hotspot sites of the S-RBD, which are likely to contribute to cell fusion through ACE2.^29^ In the end, all the best binders were then screened through the QSAR model for their biological activities, including mutagenicity, sensitivity, biodegradability, toxicity, and carcinogenicity.

Among the screened antiviral drugs to treat COVID-19, hydroxychloroquine and favipiravir, followed by fosamprenavir and abacavir, exhibited the maximum number of H-bonds with the RBD region of the spike protein. Based on the media debates about the efficacy of the two popular anti-malarial agents chloroquine and hydroxychloroquine against SARS-CoV-2, hydroxychloroquine displayed four potential H-bond interactions, with minimal IE ( − 7.99 kcal/mol) and BA ( − 3.56 kcal/mol) compared with chloroquine: two H-bonds, IE of − 3.56 kcal/mol, and BA of − 25.82 kcal/mol. These results are in line with the recently published *in silico* docking study conducted by Amin et al. (2020) using BIOVIA Discovery Studio.^35^ Notably, similar results were also obtained by the use of the AutoDock simulations platform.^36^ It is suggested that hydroxychloroquine increases the pH within intracellular vacuoles and alters the processes of protein conformational change and its fusion with the cell membrane ^37^. Some previously published data showed that hydroxychloroquine effectively inhibited the entry, transport, and post-entry stages of SARS-CoV-2.^38^ Another study found hydroxychloroquine to be a more potent agent than chloroquine at inhibiting SARS-CoV-2 *in vitro*, concordant with our observations ^39^.

On the other hand, favipiravir extended four H-bond interactions with the RBD region, with IE and BA scores of − 8.34 kcal/mol and − 45.23 kcal/mol, respectively. Other docking studies identified favipiravir with a potential BA^40–42^ to protease and RdRp proteins of SARS-CoV-2 using molecular docking software's such as AutoDockVina suite, ^43^ and Autodock4 methods ^44^. Functionally, favipiravir exhibits its antiviral property by targeting the catalytic domain of RNA-dependent RNA polymerase and interrupting viral RNA replication.^45^ It is currently administered to treat COVID-19 patients and has shown superior treatment efficacy (recovery rate: 71.43%) in a randomized control trial (ChiCTR200030254) ^42^.

Fosamprenavir and abacavir exhibited the maximum number of three H-bond interactions with the RBD region and further extended interactions with other residues in the spike. Fosamprenavir, an analog of Amprenavir, was also reported in *in silico* studies targeting the protease and RdRP regions of SARS-CoV-2 S ^46^. Fosamprenavir suppresses the cleavage of a polyprotein into multiple functional proteins, blocking the reverse transcriptase enzyme needed for HIV replication.^47^ On the other hand, abacavir, the potential binder in our study, was not proposed for the treatment of Covid-19. The rest of the selected molecules, such as penciclovir, lopinavir, chloroquine remdesivir, ribavirin, nafamostat, and nitazoxanide participated in fewer potential interactions. Thus, our study confirms the superiority of hydroxychloroquine, favipiravir, fosamprenavir, and abacavir over currently available drugs for their better interactions with S-protein *in silico* in terms of H-bonds, IE, and BA.

A screen of natural compounds resulted in identification of glycyrrhizin, nepitrin, punicalagin, EGCG, theaflavin, silibinin, galuteolin, 7- galloytriacetone, procyanidin, and catechin as potential binders to the S-RBD. Glycyrrhizin and nepitrin were the top two among them, participating in 12 and 10 H-bonds, respectively. Numerous pharmacologic activities, such as anti-inflammatory, antiviral, antitumor, and hepatoprotective properties, have been attributed to glycyrrhizin ^48^. In 2005, Hoever et al. demonstrated glycyrrhizin's inhibitory action on SARS-coronavirus (SARS-CoV) replication *in vitro.* ^49^ It was demonstrated that glycyrrhizin possessed antiviral effects against SARS-CoV *via* targeting S-protein and blocking the binding of S-protein to ACE2 in a dose-dependent manner.^50,51^ Neptrin is also known to possess significant anti-inflammatory activity in acute and chronic modes of inflammation.^52^ Punicalagin has been reported as responsible for virus internalization by various investigations and is currently provided as supplementary treatment with beneficial effects in COVID-19 therapy.^53,54^ EGCG from green tea polyphenols was reported recently to block the spread of SARS-CoV-2 by preventing its fusion with host cells via glucose-regulating protein 78.^55^ Theaflavin isolated from black tea polyphenols functioned as a potential spike protein blocker against SARS-CoV-2.^56^ Interestingly, none of the natural compounds interacted with the potential N-linked vs. O-linked glycosylation site but interacted with hotspot residues. Binding to these hot spot residues on the S-RBD surface is likely to contribute a significant portion of the binding energy to ACE2 and is thus expected to reduce the conformational changes required for membrane fusion.^29^

A virtual screening strategy to screen candidate natural compounds inhibitors against SARS-CoV-2 spike protein resulted in 25 lead binders with a maximum number of H-bonds and a minimum IE and BA score. These 25 lead binders included NPC105283, NPC107966, NPC105800, NPC121573, NPC130586, NPC100612, NPC103398, NPC129417, NPC1291, NPC100251, NPC114659, NPC131747, NPC129624, NPC114505, NPC131874, NPC11847, NPC102851, NPC100936, NPC132636, NPC13193, NPC126779, NPC119794, NPC11551, NPC130536, and NPC120099. Among them, two of the top compounds, NPC105283 and NPC107966, participated in 11 H-bond interactions with an average IE of − 2.77 kcal/mol and a BA of − 56.6 kcal/mol. Subsequent biological activity analysis of these compounds in the NPASS database revealed that NPC105283 and NPC107966 have radical scavenging activity.^24^ Surprisingly, we did not obtain any indication of these compounds’ antiviral activities in the NPASS database. Hence, these screened natural inhibitors did not interact with any of the potential glycosylation or hot spot residues but significantly inhibited RBD SD1 of the new anti- SARS-CoV-2 spike protein drugs. These interactions with spike protein may interfere with the refolding of the spike, therefore, inhibiting the viral infection process.^40^ Due to the presence of enormous structural and chemical diversity, the availability of more chiral centers, and relative biosafety, natural compounds are considered an excellent source of drugs for the treatment of several diseases, including viral infections.

Collectively, our identified drug-like molecules are found to make strong H-bonds with known crucial active residues; thereby, these interactions are supposed to reduce S-protein activity, which in turn eases the virulence activity.^35^ Our study warrants further *in vitro* activity experiments with SARS-CoV-2 infected cell-based assays, followed by individual protein targets to evaluate these potential compounds. In cell-based screening with an effective concentration (EC50) in the 2–20 μM range, these computationally selected drug-like molecules may guide the choice for further downstream experiments and validation in small animal models.^57^ To summarize, our findings probed potential inhibitors based on natural compounds that can be used in the combination of drugs as a complex to act as an inhibitor of SARS-CoV-2 to potentiate effects of drugs with originally moderate benefit.

## Conclusion

In conclusion, we identified 45 compounds with potential inhibitory activity for the SARS-CoV-2 RBD region: 10 currently prescribed drugs, 10 natural antiviral compounds, and 25 natural compounds. Among the 10 currently prescribed drugs, hydroxychloroquine and favipiravir exhibited the maximum number of H-bonds with the RBD region. Of the natural antiviral drugs, 10 compounds arise as lead candidates due to their high H-bond activities and low interaction energies. Glycyrrhizin and nepitrin topped among the natural antiviral drugs by extensively occupying the RBD region of the spike protein. Further, among the screened natural compounds from the NPASS database, 25 molecules significantly occupied the RBD SD1 region of the new anti- SARS-CoV-2 spike protein crucial for cell fusion ACE2. So, these drugs with a combination therapy approach, drugs with dose-limiting toxicity, can be administered as monotherapies, thereby reducing toxicity and enhancing the synergistically therapeutic efficacy of the drug molecule. However, drug combination therapy using multiple drugs at low concentrations was also shown to effectively block viral infection *in vitro.* ^58,59^ Therefore, synergistic drug combinations can be mainly useful for drug repurposing.^60^ As for SARS-CoV-2, a multitarget treatment approach with synergistic drug combinations containing different mechanisms of action, including inhibition of viral entry and replication and inhibition of host immune responses, maybe a useful and practical therapeutic strategy for the treatment of severe COVID-19.

### Conflict of interest

All authors do not have any conflict of interest.

### Authors’ contributions

Conceived and designed the experiments: SMM, FBS, HMY

Funding: AAT, HMY

Performed the experiments: SMM

Analyzed the data: SMM

Wrote the manuscript: SMM, FBS, HMY

All authors revised the manuscript and agreed with the final submitted version.

### Funding

This study was supported by funding from Qatar University, grant # QUCG-BRC-20/21-1.

## Figures and Tables

**Table 1:** List of the best binders among the currently prescribed drugs ranked on the basis of H-bond interactions with the RBD region of the SARS-CoV-2 spike protein

S. No.	Derivative Name	Chemical Structure	Interaction Energy(Kcal/mol)	Number of H-Bonds	Residue Interactions	Binding Affinity(Kcal/mol)
1	Hydroxychloroquine	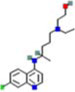	− 7.64	4	ASP428, THR430(2), PHE515	− 45.65
2	Favipiravir	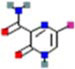	− 8.34	4	ASP428, THR430(2), PHE515	− 45.23
3	Penciclovir	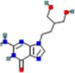	− 5.39	3	THR430, SER574, PHE515	− 35.86
4	Lopinavir	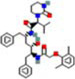	− 6.45	2	LYS386 (2), ASN978, ILE980, ASP979, SER982	− 25.82
5	Chloroquine	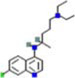	− 5.34	2	SER514, LEU517	− 30.75
6	Remdesivir	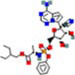	− 3.86	2	SER982, ASP979	− 17.32
7	Ritonavir	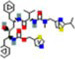	− 1.11	2	SER514(2)	− 10.54
8	Ribavarin	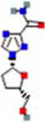	− 1.64	2	LEU517, ASP979	− 12.21
9	Nafamostat	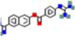	− 2.34	1	PRO463	10.33
10	Nitazoxanide	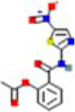	− 2.42	2	LEU517(2)	9.31
11	Oseltamivir	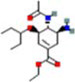	− 1.14	2	LEU517(2)	7.31
12	Chlorphenamine	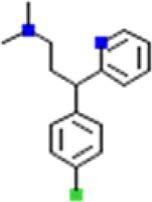	0	0	0	0

**Table 2:** List of the best binders among the antiviral drugs ranked on the basis of H-bond interactions with the RBD region of the SARS-CoV-2 spike protein

S. No.	Derivative Name	Chemical Structure	Interaction Energy(Kcal/mol)	Number of H-Bonds	Residue Interactions	Binding Affinity(Kcal/mol)
1	Fosamprenavir	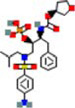	− 5.66	5	SER383, ASP389, ASN542, SER982, ARG983	− 22.66
2	Atazanavir	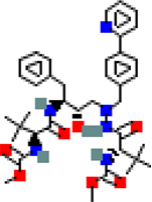	− 4.21	3	GLY381(2), ILE980	− 17.68
3	Abacavir	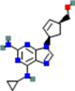	− 6.88	5	PHE543, ASP389, ASN542, LEU984, SER982	− 18.85
4	Indinavir	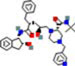	− 7.52	3	GLY381(2), ASP428	− 18.74
5	Raltegravir	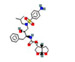	− 2.99	4	ARG357(2), TYR396, TYR170	− 15.85
6	Darunavir	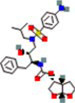	− 4.95	1	SER982	− 14.86
7	Tipranavir	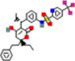	− 3.22	1	ASP979	− 13.42
8	Elvitegravir	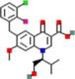	− 6.95	2	ASV542, ASP389	− 14.86
9	Ritonavir	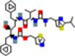	− 4.55	2	GLY381, ASP979	− 12.86
10	Saquinavir	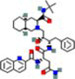	− 5.87	1	LYS356	− 14.27

**Table 3:** List of best binders among the 100 natural antiviral compounds ranked on the basis of H-bond interactions with the RBD region of the SARS-CoV-2 spike protein

S. No.	Derivative Name	Functional Groups	Interaction Energy(Kcal/mol)	Number of H-Bonds	Residue Interactions	Binding Affinity(Kcal/mol)

1.	Glycyrrhizin	**Saponin**	− 6.32	12	VAL382, TYR380(2), THR430(4), GLY431, ASP428(2), ASP427(2)	− 42.55

2.	Nepitrin	**Flavonoid**	− 6.32	10	ASP428, THR430(2), PHE515, LYS386(2), ARG983, SER383, SER982(2)	− 47.85

3.	Punicalagin	**Ellagitannin**	− 5.5	23	PHE429, DER514(2), GLU516(2), TYR396, ARG355, PHE464, TYR200, ILE197, LYS202, ASN196, LEU229, ASP228(2)	− 50.34

4.	EGCG	**Flavan-3-ol**	− 6.32	12	LYS386(2), ASP389(3), ASN542(2), PHE543, SER982, ILE980, ASP979(2)	− 52.15

5.	Theaflavin	**Flavan-3-ol**	− 4.35	13	GLY545, THR547, LYS386, ASN389(4), ASN542, ASN978, SER982(2), LEU981, ARG983	− 56.55

6.	Silibinin	**Flavonolignans**	− 6.32	13	LYS386, THR430(3), ASP428, LEU517, HIS519, SER982(3), ARG983(3)	− 53.46

7.	Galuteolin	**Flavonoid**	− 3.21	10	ASP428, THR430(2), PHE515, LYS386, SER383, ASP979, LEU984, SER982, LEU981	− 47.85

8.	Galloytriacetone	**Flavonoid**	− 3.21	8	LYS386, SER383(4), PHE515, SER982, ARG983	− 49.86

9.	Procyanidin	**Flavonoid**	− 3.21	7	ASP428, THR430(2), PHE515, LYS386(2), HIS519,	− 56.85

10.	Catechin	**Flavan-3-ol**	− 5.55	7	LYS386, THR430(3), ASP428, LEU517, HIS519,	− 53.01


**Table 4:** List of the 25 best binders ranked on the basis of H-bond interactions with the RBD region protein of the SARS-CoV-2 spike protein

S. No.	Derivative Name	Interaction Energy(Kcal/mol)	Number of H-Bonds	Residue Interactions(RBD region)	Binding Affinity(Kcal/mol)

1.	NPC105283	3.32	11	TYR351, SER349(3), PHE347, ARG346(3), LYS356, TYR451	− 57.45

2.	NPC107966	− 2.22	11	PRO412, TYR508(2), GLY404, GLN414, LYS378(2), SER378(3)	− 55.75

3.	NPC105800	− 2.36	10	LYS356(3), GLU340(2), SCR359(2), ARG355, ASN3545, ARG346	− 54.97

4.	NPC121573	− 1.22	10	ARG408, ILE410, TYR380(2), THR376(2), SER375, PHE377(3)	− 48.85

5.	NPC130586	2.36	10	TYR380(2), ILE410, THR376(2), GLY404(2), ARG408, ASP405, TYR508	− 51.13

6.	NPC100612	3.32	10	SER359(2), ARG357, GLU340, ASN354, LYS356, ASN354, TYR451(2), ASP442	− 50.23

7.	NPC103398	2.36	9	ASP428, THR430(3), PHE515(2), LEU517(2), HIS519	− 54.76

8.	NPC129417	− 2.86	9	PHE515, LEU517, THR430(2)ASP428(5), ASP427(2)	− 51.64

9.	NPC1291	− 4.13	9	LEU517(2), HIS519, PHE515, THR430(3), ASP428(2)	− 47.64

10.	NPC100251	− 2.86	8	LYS378(5), TYR380, ARG408, VAL407	

11.	NPC114659	− 4.52	8	ARG355, LYS356(3), GLU340, ARG346(2), THR470	− 53.86

12.	NPC131747	− 2.32	8	ASP427(2), ASP428, THR430(3), GLY381, PHE515	− 48.87

13.	NPC129624	1.87	8	HIS519, LEU517(2), GLY381, THR430(2), ASP428(2)	− 51.15

14.	NPC114505	− 2.75	8	LYS378(2), ASP427, ILE410, VAL407, ARG408, TYR380(2)	− 50.46

15.	NPC131874	− 5.21	8	HIS519, LEU517, THR430(3), ASP428(3)	− 47.25

16.	NPC11847	1.87	7	PHE377, LYS378(3), TYR380, VAL407, GLN414	− 52.87

17.	NPC102851	1.87	7	TYR380, THR430, ASP427, LYS378, PRO412, ASP427(2)	− 51.65

18.	NPC100936	2.63	7	LYS378, TYR380(2), PRO412, ASP428, ASP427	− 53.87

19.	NPC132636	− 1.85	7	LYS378(2), TYR380(3), ILE410(2)	− 52.70

20.	NPC13193	4.25	7	ASP428, PRO412(2), LYS378(2), ARG408(2)	− 53.85

21.	NPC126779	− 2.75	7	TYR369, LEU368, PHE374, PHE377, SER375(3)	− 49.85

22.	NPC119794	− 2.86	6	ASP427, LYS378, PHE377, GLN414, ARG408, VAL407	− 53.76

23.	NPC11551	− 2.22	6	ASP428(2), TYR380, ILE410, ARG408, LYS378	− 47.85

24.	NPC130536	− 2.75	6	TYR380, VAL407, GLN414, GLN414, ARG408(2)	− 48.97

25	NPC120099	− 8.61	6	ASP428(2), TYR380(2), GLN414, ARG408	− 54.97

